# N-Butanol and Aqueous Fractions of Red Maca Methanolic Extract Exerts Opposite Effects on Androgen and Oestrogens Receptors (Alpha and Beta) in Rats with Testosterone-Induced Benign Prostatic Hyperplasia

**DOI:** 10.1155/2017/9124240

**Published:** 2017-12-11

**Authors:** Diego Fano, Cinthya Vásquez-Velásquez, Cynthia Gonzales-Castañeda, Emanuel Guajardo-Correa, Pedro A. Orihuela, Gustavo F. Gonzales

**Affiliations:** ^1^Laboratory of Endocrinology and Reproduction, Department of Biological and Physiological Sciences, Faculty of Sciences and Philosophy, Universidad Peruana Cayetano Heredia, San Martín de Porres, Peru; ^2^Research Circle in Plants with Effects in Health, Universidad Peruana Cayetano Heredia, San Martín de Porres, Peru; ^3^Laboratory of Immunology of the Reproduction and Centro para el Desarrollo en Nanociencia y Nanotecnologia (CEDENNA), Universidad de Santiago de Chile, Región Metropolitana, Chile

## Abstract

Benign Prostatic Hyperplasia (BPH) affects, worldwide, 50% of 60-year-old men. The Peruvian plant red maca* (Lepidium meyenii)* inhibits BPH in rodents. This study aimed to determine the effects of methanolic red maca extract and its n-butanol and aqueous fractions on expression of androgen and oestrogen receptors in rats with testosterone enanthate-induced BPH. Thirty-six rats in six groups were studied. Control group received 2 mL of vehicle orally and 0.1 mL of propylene glycol intramuscularly. The second group received vehicle orally and testosterone enanthate (TE) (25 mg/0.1 mL) intramuscularly in days 1 and 7. The other four groups were BPH-induced with TE and received, during 21 days, 3.78 mg/mL of finasteride, 18.3 mg/mL methanol extract of red maca, 2 mg/mL of n-butanol fraction, or 16.3 mg/mL of aqueous fraction from red maca. Treatments with red maca extract and its n-butanol but not aqueous fraction reduced prostate weight similar to finasteride. All maca treated groups restored the expression of ER*β*, but only the aqueous fraction increased androgen receptors and ER*α*. In conclusion, butanol fraction of red maca reduced prostate size in BPH by restoring expression of ER*β* without affecting androgen receptors and ER*α*. This effect was not observed with aqueous fraction of methanolic extract of red maca.

## 1. Introduction

Benign Prostatic Hyperplasia (BPH) is a disease of high relevance since, worldwide, it affects 50% of men aged 60-year-old [1], reaching levels of 90% in men older than 80 years [2]. Clinically, it is characterized by presenting lower urinary tract symptoms (LUTS), nocturia, sepsis, irreversible bladder failure, and even death [1].

The etiology is still unclear, but it is largely accepted that sex hormones are fundamental. Androgen receptor (AR) has received special attention, due to its effects on prostate growth signaling pathways; for instance, androgens promote the expression of Epidermal Growth Factor, Keratinocyte Growth Factor, and Insulin-like Growth Factor [3, 4].

Although oestrogens and their receptors *α* and *β* (ER*α*, ER*β*) are less studied than AR, it is evident that they also play a significant role on normal prostate growth [5, 6]. ER*α* activation is related to the inflammatory and proliferative response whereas ER*β* activation has an antiproliferative and proapoptotic response, counteracting AR and ER*α* effects.

Nowadays, treatments for BPH include finasteride, tamsulosin or, in severe cases, transurethral resection of the prostate. Although they ameliorate LUTS symptoms and, in case of finasteride, decrease prostatic volume, they are not exempt of side effects like hematuria, loss of sexual desire, abnormal ejaculation, among others [7]. Evidently, these side effects significantly alter quality of life of men with BPH. For such reasons, scientists are looking for better options of treatments including natural products [8].

A particular outcome of this interest was the discovering of a Peruvian plant, red maca (RM,* Lepidium meyenii*), as a useful agent against BPH by reducing prostate size in rats with testosterone enanthate- (TE-) induced BPH without affecting seminal vesicles, being better when compared with finasteride treatment [9–14].

In 2002, Piacente et al. [15] studied methanolic extract of maca and its n-butanol and aqueous fractions, establishing that butanol fraction contains alkaloids as (1R,3S)-1-methyltetrahydro-beta-carboline-3-carboxylic acid (MTCA). These authors suggest that this compound could be harmful. This was confronted by data from others reporting that MTCA is a natural constituent of many plants and no toxicity is found on consumption of such whole plants. This suggests that as multicomponent, MTCA may lose adverse drug action [16].

Recently, two studies with n-butanol fraction and aqueous fraction of methanolic extract of red and black maca showed evidence that these fractions are not toxic. In mice, aqueous fraction of black maca increased sperm count, an effect not observed with n-butanol fraction [17]. The second study with red maca extract and fractions administered during 90 days demonstrated no side effects on liver and kidney function [18].

The present study aimed to assess the effect of methanolic RM extract and its n-butanol and aqueous fraction in TE-BPH rat prostate and on AR, ER*α*, and ER*β* expression levels.

## 2. Materials and Methods

### 2.1. Experimental Animals

Thirty-six three-month-old Holtzman strain male rats* (Rattus norvegicus)* were acquired from the Universidad Peruana Cayetano Heredia Animal House. The rats had an initial body weight of 300 ± 1.49 g (mean ± SEM). They were allocated in animal cages in a rate of 6 rats per cage. The rats were kept at 25°C with periods of light/darkness of 12 hours inside the experimental room. They received food and water ad libitum.

Animals were maintained and managed according to the United States National Institute of Health “Guide for the Care and Use of Laboratory Animals” [19].

### 2.2. Experimental Design

This is an experimental study with a follow-up of 3 weeks in which rats received two doses of testosterone enanthate to induce BPH, and then they were treated with vehicle, finasteride, RM methanolic extract, or its fractions (aqueous or n-butanol) during 21 days. All treatments were daily administered by oral inoculation.

The study included six groups of six rats each (*n* = 36). Group 1 was control in which water was inoculated as vehicle (VH) of RM methanolic extract (RM) and propylene glycol (inert oil) was intramuscularly injected. Except for Group 1, all groups received two intramuscular injections of testosterone enanthate (Testoviron®, BAYER, Lima, Peru) at day 1 and at day 7 of treatment, resulting in a total of 50 mg of TE for each rat. Group 2 (TE) was treated with water by gavage needle with vehicle only. Group 3 (TE + F) was treated with 0.6 mg/kg of finasteride. Group 4 (TE + MetOH) received 36.1 mg of methanolic RM extract; the fifth group (TE + ButOH) was treated with a daily dose of 4.0 mg of butanol fraction, and the last group (TE + Aq) was treated with a daily dose of 32.5 mg of the aqueous fraction. At day 22, prostate glands were surgically removed and their weight was evaluated.

### 2.3. Red Maca Methanolic Extract and Its Fractionation

Dried RM was obtained from Junin at 4200 meters above sea level, in the Peruvian central Andes. The plant was authenticated by Biol. Camilo Díaz, a botanist from the Pharmaceutical Sciences Section of the Faculty of Sciences and Philosophy at the Universidad Peruana Cayetano Heredia.

Red maca methanolic extract and its n-butanol and aqueous fraction were prepared as established by Piacente et al. in 2002 [15]. In brief, one kilogram of dried red maca hypocotyls was ground, followed by 72 hours of maceration in 2 L of methanol with constant agitation. The liquid is filtered and methanol excess is removed in a rotary evaporator. Under this procedure, 121.9 g of methanolic extract was obtained. An amount of 8.25 g of the resulting extract was collected and dissolved in a butanol/water (1 : 1) solution and left to settle down for 72 hours in a separatory funnel. Each phase was separated and solvent excess was taken out in the rotary evaporator.

### 2.4. Euthanasia

Euthanasia of experimental rats was performed using 1 mL of Halatal® (Montana S.A., Lima, Peru) by intraperitoneal injection. After that the rats were disposed in the cages until cardiac pulse and respiration rhythm stopped.

### 2.5. Histological Evaluation

One of the two lobes of the ventral prostate was fixed in formaldehyde for three days and then in ethanol 75%. The organs were paraffin-embedded. Paraffin blocks were cut with a microtome at 6 *μ*m thickness. Half of the sections obtained were stained with Hematoxylin and eosin. Slides were observed with a compound microscope (Leica DM1000).

The other half was used for IHC staining with anti-AR (sc-816), mouse anti-ER*α* (sc-787), and goat anti-ER*β* (sc-6821) (Santa Cruz Biotechnology, Dallas, USA). In a 1 : 50 dilution, primary antibody was incubated overnight at 4°C. Prior to primary antibody incubation, slides were soaked in Citrate buffer (11.5 mM sodium citrate, 0.5 mM Tween 20) at 100°C for 40 minutes to retrieve the antigen. Endogenous peroxidase deactivation was done by submerging the slides in peroxide 30%/methanol solution (1 : 5).

The secondary antibody recognition and staining was performed with Histostain-Plus IHC Kit, HRP, broad spectrum® (Thermo Fisher Scientific, USA). To assess the expression of the different receptors, a ratio within positive nuclei/total nuclei was elaborated. Nuclei visualization and count was performed with a compound microscope (Leica DM1000). Four visual fields per slide were assessed and three slides per animal (prostate lobe) were evaluated.

### 2.6. Western Blotting Assay

Whole organ tissue was mechanically lysed with TissueTearor® (Biospec Products). The homogenized prostate was resuspended in RIPA buffer (10 mM Tris-HCl pH 8.0; 1 mM EDTA; 1% NP-40; 0,1% sodium deoxycholate; 140 mM NaCl; 1 mM Complete 20®) and centrifuged at 10000 RPM for 15 minutes at 4°C.

Total proteins were quantified with Bradford assay method. Samples were stored at −80°C until use. Thirty *μ*g of total protein from each sample was resuspended in loading buffer (0.0625 M Tris/HCl pH 6.8, SDS 1%, *β*-mercaptoethanol 2.5%, glycerol 10%, and bromophenol blue 0.0001%) and heated for 10 minutes at 60°C. Afterwards, a SDS-PAGE was performed in 10% and 15% gels. Proteins were blotted with TRANS-BLOT® SD SemiDry Transfer Cell (BioRad) into a nitrocellulose membrane. Membranes were blocked overnight with 10% skimmed milk at 4°C. All washes were done with TTBS buffer.

Corresponding primary antibody was diluted in TTBS buffer (anti-AR 1/5000, anti-ER*α* 1/10000, and anti-ER*β* 1/5000); for normalization, anti-*β*-actin antibody was used (1/10000, sc-47778, Santa Cruz Biotechnologies). Primary antibody incubation had a length of 2 hours, following 1 hour of secondary antibody incubation (ab 97409, Abcam) each at room temperature. Target proteins were detected by chemiluminescence in a radiographic film. Optical density quantification was performed using Image J software (http://rsb.info.nih.gov/ij/).

### 2.7. Statistical Analysis

The statistics software STATA 12.0 (StataCorp LLC, Texas) package was used. Variance homogeneity was evaluated by Bartlett's test and data normal distribution by Shapiro-Wilk test. If the variables were homogeneous, differences between groups were assessed by ANOVA test. If *F* value was significant (*p* < 0.05), to distinguish which groups were different, Scheffé's test was applied. If data have not normal distribution, nonparametric test Kruskal-Wallis was used and post hoc Dunn test.

## 3. Results

### 3.1. Effects of Different Treatments on Prostate Weight, Histomorphology, Stromal Area, and Epithelium Height

**Figure 1:**
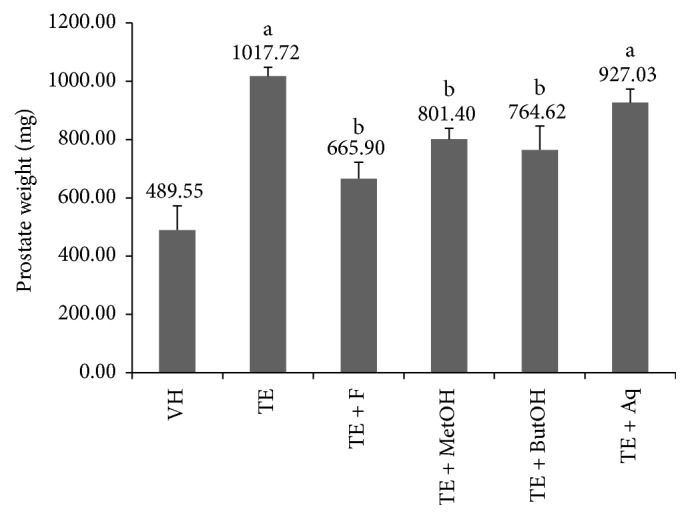
Mean prostate gland weight in all experimental groups. a: *p* < 0.05 compared to VH. b: *p* < 0.05 compared to TE.

As expected, ET group (1017.72 ± 29.53 mg) had a greater prostate weight compared to VH (*p* < 0.05). Prostate weight in TE + F, TE + MetOH, and TE + ButOH showed similar values compared to VH group (489.55 ± 86.63 mg, *p* > 0.05). Surprisingly, TE + Aq group prostate weight (927.03 ± 46.48 mg) was not different compared to TE (*p* > 0.05) (Figure 1).

**Figure 2:**
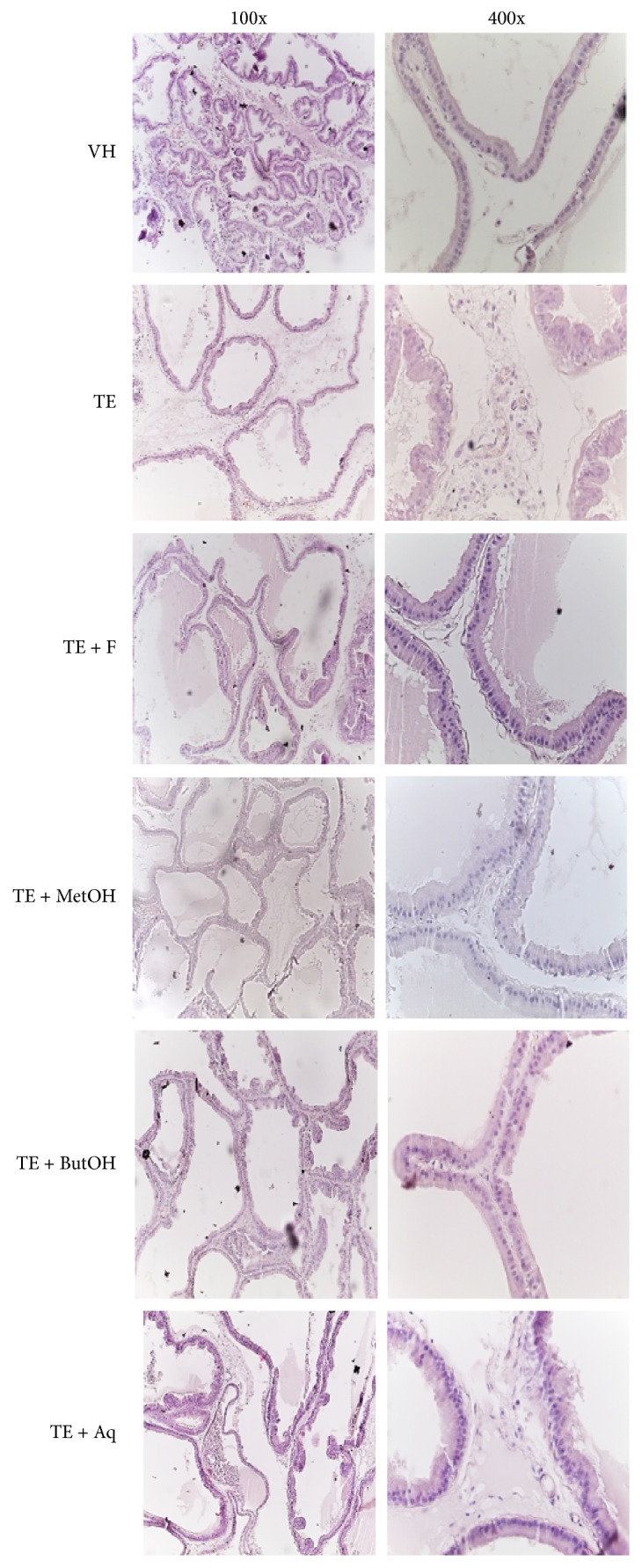
Microphotographs of prostate tissue in all treatments at two different magnifications: 100x and 400x.

As observed in a previous study [20], in the BPH model the prostatic acini lost the characteristic inner projections of the epithelium. This alteration also occurred in the treatment groups; nonetheless, there is an evident increase of both epithelial cells height and stromal area in TE and TE + Aq groups (Figure 2).

**Figure 3:**
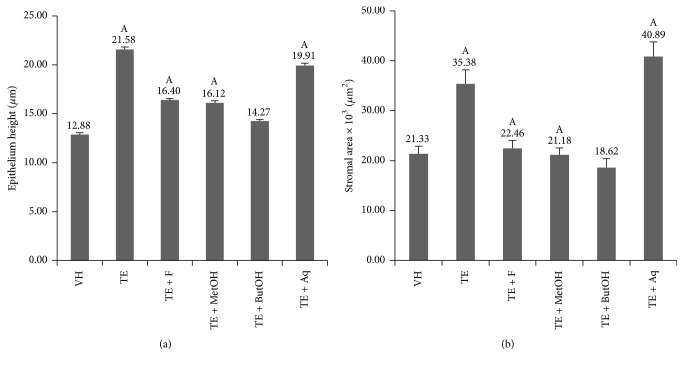
Epithelium height values (a) and stromal area (b) values in the different treatments. A: *p* < 0.05 compared to VH.

Prostate weights, epithelial height, and stromal area values were higher in both TE and TE + Aq groups compared to VH (*p* < 0.05) and with respect to the other treatments (*p* < 0.05) (Figure 3(a)); the other groups also showed an increase in epithelium height compared to VH (*p* < 0.05), but similar stroma area (Figure 3(b)).

### 3.2. Effect of Different Treatments on Androgen Receptor, Estrogen Receptor-*α*, and Estrogen Receptor-*β*

**Figure 4:**
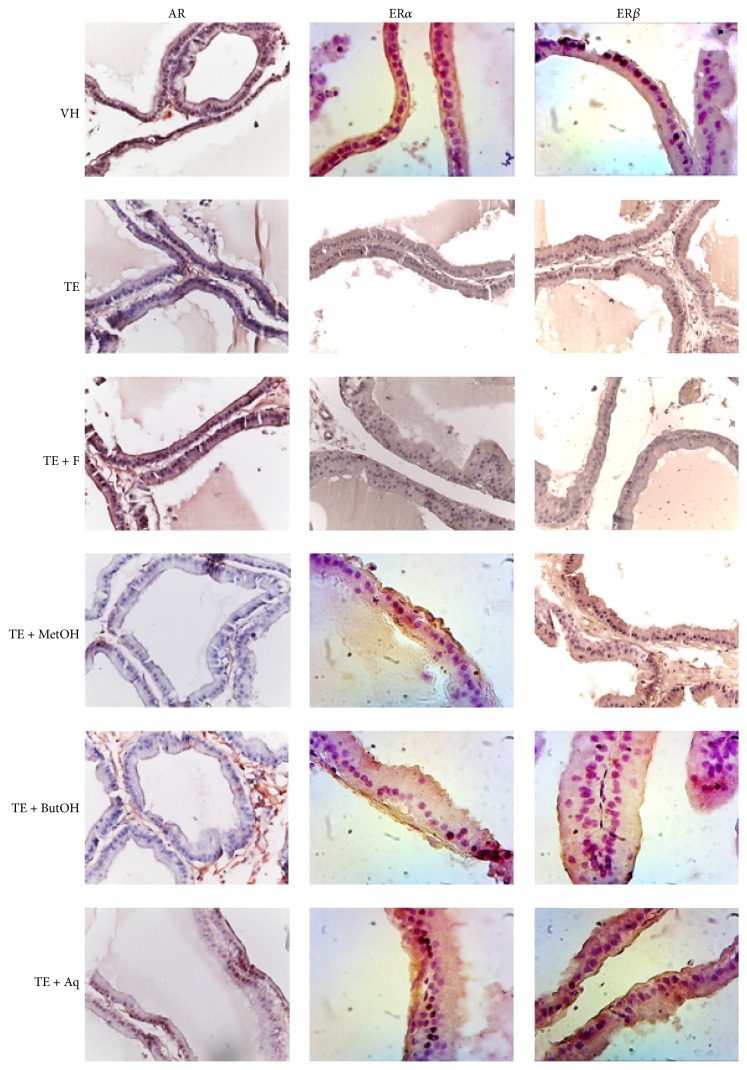
Microphotographs of IHC assays for AR, ER*α*, and ER*β* at a 400x magnification.

Different receptors expression was assessed in the epithelium of prostatic acini. The brown positive mark in some cases is weak, being a brown dot inside the nucleus, while, for example, in VH and TE + MetOH group ER*β* nuclear expression is notoriously greater (Figure 4).

Ratio values indicate that the expression of AR and ER*β* is weaker in TE group (*p* < 0.05), while for ER*α* it is similar compared to VH and the rest of groups (*p* > 0.05), except for TE + Aq, which exhibit an overexpression of AR and ER*α* (*p* < 0.05), while for ER*β* it shows similar ratio compared to TE group (*p* > 0.05).

**Figure 5:**
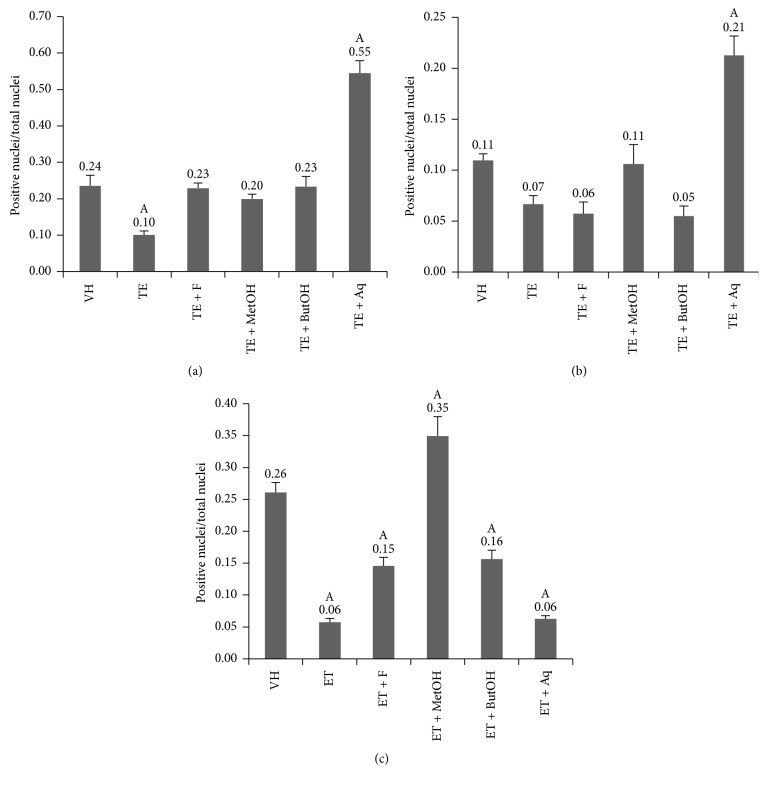
Ratio of positive nuclei/total nuclei for AR (a), ER*α* (b), and ER*β* (c). A: *p* < 0.05 compared to VH.

RM methanolic extract and its n-butanol fraction restore the expression of AR. There is a similar trend in the case of the n-butanol fraction to equate ER*β* levels; notwithstanding TE + MetOH extract of RM showed a greater ER*β* expression levels (*p* < 0.05) as seen in Figure 5.

**Figure 6:**
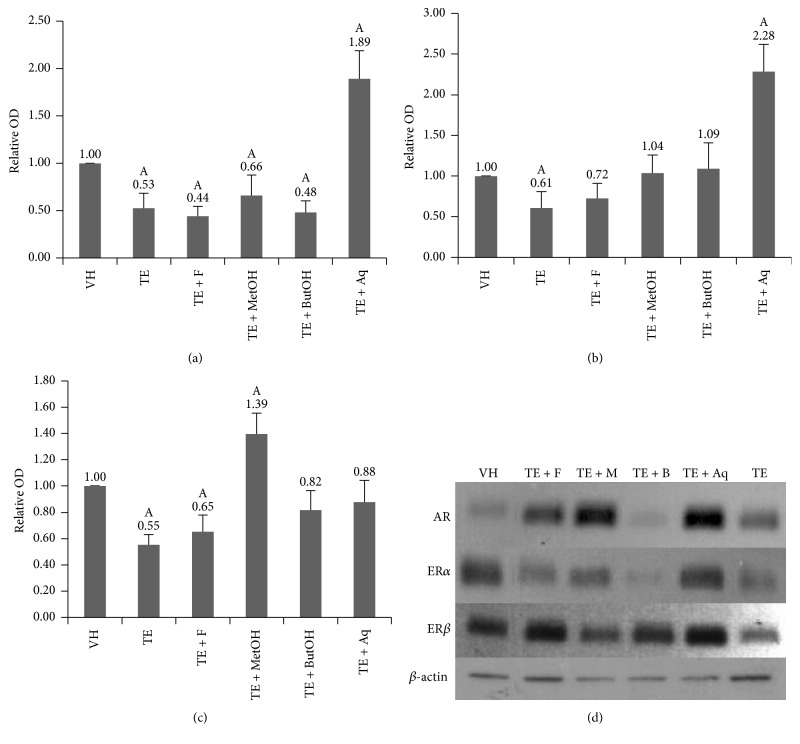
Relative optical density (OD) of AR (a), ER*α* (b), and ER*β* (c) in regard to VH optical density values and detected protein with western blot assay. Proteins detected in the different groups: TE + MetOH (TE + M) and TE + ButOH (TE + B) by western blotting (d); A: *p* < 0.05 compared to VH.

At a protein level, similar results were obtained compared to IHC assays, showing an increase of AR and ER*α* (89% and 128% with respect to VH value, resp., *p* < 0.05) in the group treated with aqueous fraction of red maca and a greater ER*β* expression (39% higher compared to VH, *p* < 0.05) in TE + MetOH group. Surprisingly, the aqueous fraction treatment displayed a restoring effect in a similar fashion as n-butanol fraction treatment on ER*β* expression, becoming similar to that observed in the VH group (*p* > 0.05) as shown in Figure 6.

## 4. Discussion

The present study aimed to assess the biological effects of red maca methanolic extract and its n-butanol (alkaloidal) and aqueous fractions on testosterone enanthate-induced BPH in rats determined by prostate gland weight, histological evaluation (stromal area and epithelium height), and the three main hormone receptors (AR, ER*α*, and ER*β*) genetic expression. These approaches will allow comprehending the action mechanism that red maca uses to reverse BPH and also contributing to decipher the long-unknown and unclear etiology of BPH despite of scientific community efforts.

The beneficial effect of RM in reducing BPH prostate weight was previously demonstrated [9]. Thereafter, a contribution of inflammation was observed as a way to produce BPH and that red maca has anti-inflammatory properties [14]. Wu et al. on 2012 [21] established that prostatic inflammation is related to an increase of AR expression levels, in which activation interferes with apoptotic processes [22–24] and promotes recruitment of macrophages infiltrates [2, 25].

On the other hand, the role of ER*α* and ER*β* is not fully understood. Although it has been established that the activation of the firsts leads to proliferative and antiapoptotic processes, the second ones have antiproliferative and proapoptotic effects [4, 26, 27]; nonetheless, it is not clear under what circumstances the activation of one of the two kinds of receptors occurs despite having a similar affinity to estradiol [28].

It is evident that the hormonal component (understanding it as hormone, receptor, and hormone-receptor interaction) contributes significantly to the development of the disease. This factor and the inflammatory one are not acting alone, but synergically.

One way to assess the contribution of the different hormonal receptors in the development of the disease is by evaluating their genetic expression. Two recent studies [20, 29] have reported the change of AR, ER*α*, and ER*β* expression due to testosterone propionate intramuscular injections and castration in a rat model; nonetheless, their results are not similar to each other and also different from ours.

Our data shows that, at tissue level, AR and ER*β* expression levels decrease in the BPH model, while ER*α* levels are maintained similar to those in non-BPH rats (VH group). At a protein level, by western blot analysis, all receptors are significantly diminished in the TE group compared to VH. These different outcomes might be due to the experimental models used. In our case, only two intramuscular injections of testosterone enanthate were enough and no castration was needed, so the hypothalamic-hypophyseal-gonadal axis is maintained. Two doses of TE in days 1 and 7 are enough to induce BPH as previously reported, lasting even at day 56 without more injections since day 7 [11, 12, 30], demonstrating the usefulness of this model.

Exogenous testosterone injections would initially provoke an increase of intraprostatic testosterone levels and thus a higher activity of the enzyme 5*α*-reductase and hence an accumulation of DHT, leading to a deregulation in proliferation/apoptosis balance, by the expression of androgen-dependent growth factors [31, 32] and the activation of NF-*κ*B signaling pathways promoting the expression of inflammatory cytokines such as IL-1, IL-6, and TNF*α* [14, 32, 33].

Moreover, high testosterone levels augment reactive oxygen levels (ROS) along with a decrease of catalase, superoxide dismutase, and glutathione peroxidase [29, 32, 34]. High ROS levels are positively correlated with higher levels of TNF*α* and cyclooxygenase type 2 (COX-2) [28]. In a previous study [35], it was shown that high levels of COX-2 and ROS interfered with the pathways activated by ER*β*, therefore promoting proliferation in a cancer cell line. Perhaps this is the same scenario in BPH, explaining why, in TE group, ER*β* levels are significantly decreased, supported by the hypothesis that ER*β* activation reestablishes and, even, increases its expression levels [36].

The present study shows evidence of the beneficial effects of red maca on BPH being as optimal as treatment with gold standard treatment and finasteride, but, remarkably, showing that only two of the three different maca treatments (e.g., methanolic extract and the n-butanol fraction) have this effect, while the aqueous fraction is not useful to treat this disease, maintaining prostate weight, epithelium height, and stromal area in values the same as TE group.

Androgen receptor levels were severely increased in the group treated with the aqueous fraction of the methanolic extract of red maca, while the extract and its butanol fraction do not, indicating an acute androgenic effect. It should be noted that, despite coming from the same extract, they can have opposed biological effects. This phenomenon was also reported but with black maca, where the aqueous fraction but not the n-butanol one is effective to improve mice fertility by increasing sperm count [17].

Red maca presents a favorable estrogenic effect, because it reestablishes ER*β* expression levels in similar values as observed in the VH group and, in the case of the methanolic extract, was higher for about 30%. This outcome is related to a previous study, in which, red maca extract is effective in recovering bone density in ovariectomized rats by regulating ER*β* [37]. This means that red maca has estrogenic biological effects.

The estrogenic effect is related not only to ER*β* expression but also to reestablish ER*α* levels. Keeping a stable ER*α*/ER*β* ratio is important to maintain a proper balance in cell proliferation; even this ratio is considered as an important epithelial carcinomas aggressiveness indicator [37].

Aqueous fraction shows an acute increase in ER*α* levels. This, along with the higher levels of AR, stimulates prostate growth, thus explaining its ineffectiveness in reducing BPH indicators. This overexpression of ER*α* would implicate higher expression of the Proliferating Cell Nuclear Antigen (PCNA) [38], which also implies Epithelial-Mesenchymal Transition (EMT) genes activation, whose expression is exacerbated by AR activation [29], criteria which aqueous fraction fulfills.

The androgenic effect of aqueous fraction could be the cause of ER*α* overexpression, because both increased testosterone levels and AR overexpression not only interfere with ER*β* expression and signaling pathways but also stimulates ER*α* expression [29], hence favoring cell proliferation. This also explains why in the IHC assay, aqueous fraction of red maca shows lower ER*β* compared to VH.

It is still unclear what compounds present in maca are responsible for reducing BPH. Perhaps, it is due to its content of *β*-sitosterol [38], a component with antiandrogenic effects by inhibiting 5*α*-reductase isoforms, found also in saw palmetto* (Serenoa repens)*, plant that is widely reported to reduce BPH [4].

Equol is an isoflavonoid present in beans, lettuce, and, more important, cabbage, a plant belonging to the same family of maca* (Brassicaceae)*. This molecule binds specifically to DHT, thus preventing AR activation, and also it has affinity to ER*β*, and this especial combination (isoflavone + ER*β*) can downregulate androgen actions [39, 40]. Assessing equol levels in methanolic extract and its n-butanol and aqueous fraction is material for further studies.

It seems that, in the fractionation process, part of the estrogenic effect of red maca is lost, because the expression of ER-beta levels is higher compared to VH, whereas with butanolic and aqueous fractions ER-beta levels are similar to VH. Perhaps some compounds present in both fractions act synergically.

This study also contributes to the hypothesis that the butanol fraction, which is one that contains alkaloids as MTCA, in opposition to that postulated by Piacente et al. [15], is not hazardous, but beneficial for the organism.

**Figure 7:**
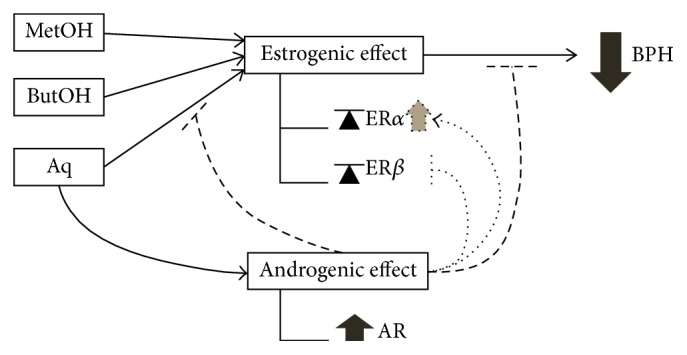
Red maca methanolic extract (MetOH) and its butanolic (ButOH) and aqueous (Aq) fractions have a positive estrogenic effect by balancing ER*α* and ER*β* expression levels, but Aq also possesses an androgenic effect increasing AR levels, and, due to this, its positive estrogenic effect and thus its action against BPH would be blocked (dashed truncated arrows), due to an acute increment of ER*α* levels and by interfering with ER*β* pathways (dotted truncated and normal arrow).

In summary, red maca methanolic extract and its butanol and aqueous fractions have a favorable estrogenic effect by reestablishing ER*α* and ER*β* levels; nonetheless, in the case of the aqueous fraction, its androgenic effect counteracts this beneficial effect, not only by promoting cell growth via AR activation, but also by increasing ER*α* levels, in which mixed actions would interfere with both ER*β* expression and signaling pathways (Figure 7).
